# Supplementary material for: S100A8/A9 Enhances Immunomodulatory and Tissue-Repairing Properties of Human Amniotic Mesenchymal Stem Cells in Myocardial Ischemia-Reperfusion Injury
Source: Int J Mol Sci. 2021 Oct 16;22(20):11175. doi: 10.3390/ijms222011175 (PMC8541313; doi:10.3390/ijms222011175)
Supplement: Supplementary file 1 [file ijms-22-11175-s001.zip › ijms-1382341-supplementary.pdf]

International Journal of
Molecular Sciences

MDPI

Supplementary Materials

Table S1. The human gene sequences of qPCR. All the primer sequences have validated the function through preliminary RT-PCR.

Primer ID	Sequences (5'→3')	TM (°C)	Product
GAPDH-F	CCATGTTGCAACCGGGAAG	53.2	NM_001256799.3
GAPDH-R	GCCCAATACGACCAAATCAGAG	54.8	
CD105-F	GATAAGGCCCAGCGCACA	52.6	NM_000118.3
CD105-R	GTTTCTGCAAGACTTGTGGGG	54.4	
CD14-F	GAAGACTTATCGACCATGGAGC	54.8	NM_000591.4
CD14-R	CGCAGCGGAAATCTTCATCG	53.8	
CD19-F	GTGGTGAAGGTGGAAGAGGG	55.9	NM_001178098.2
CD19-R	AAGGGTTTAAGCGGGGACTC	53.8	
CD34-F	GGAGAAAGGCTGGGCGAAG	55.4	NM_001025109.2
CD34-R	CACACTGGCCTTTCCCTGAG	55.9	
CD45-F	CTTGGCATTTGGCTTTGCCT	51.8	NM_001267798.2
CD45-R	TGCTGTAGTCAATCCAGTGGG	54.4	
CD73-F	TGAACGCAACAATGGCACAAT	50.5	NM_001204813.2
CD73-R	GGCCTTCTTCAGGGTGGAAC	55.9	
CD79-F	GCTGCTGCTGTTCAGGAAAC	53.8	NM_001783.4
CD79-R	GTCCAGGTTCAGGCCTTCAT	53.8	
CD90-F	GGATGGCGAGTGACTTAGGG	55.9	NM_001311160.2
CD90-R	TCTCAGTCCTGGATCGGGTT	53.8	
CDH2-F	AGGCTTCTGGTGAAATCGCA	51.8	NM_001308176.2
CDH2-R	TGCAGTTGCTAAACTTCACATTG	51.7	
CRP-F	CGGACTTTTGGTCATGAAGACAT	53.5	NM_000567.3
CRP-R	AGTGGCTTCTTTGACTCTGCT	52.4	
CCR7-F	CGTCATGGACCTGGGTATGC	55.9	NM_001301714.2
CCR7-R	GACCAGAAAGCCGATCACCA	53.8	
GATA4-F	GGGACTTGGAGGCGGC	53.6	NM_001308093.3
GATA4-R	AGCCGGGGTCCTGCTG	53.6	
GATA6-F	AGAAGCGCGTGCCTTCATC	53.2	NM_005257.6
GATA6-R	TTTCTGCGCCATAAGGTGGT	51.8	
GDF9-F	CTTGGAGGTCGTGAGGCTG	55.4	NM_001288824.4
GDF9-R	GGATGCTCCAGCTGGTCTTT	53.8	
HLADR-F	CTCCTGCATGGCAGTTCTGA	53.8	NM_001775.4
HLADR-R	TCCAAGAAACGTGGTCTGGT	51.8	
IDO1-F	AGATGTCCGTAAGGTCTTGCC	54.4	NM_002164.6
IDO1-R	AGCTATTTCCAACAGCGCCT	51.8	
IFN-F	GGAGGAACTGGCAAAAGGATG	54.4	NM_024013.3
IFN-R	GTTGCTGATGGCCTGATTGT	51.8	
IL10-F	CGAGATGCCTTCAGCAGAGT	53.8	NM_000572.3
IL10-R	GAAGAAATCGATGACAGCGCC	54.4	
IL6-F	CTCATTCTGCTCTGGAGCCC	55.9	NM_000600.5
IL6-R	CAACTGGATGGAAGTCTCTTGC	54.8	
MMP2-F	TGCCCCCATGAAGCCTTG	52.6	NM_001127891.3
MMP2-R	TACAGCTGTTGTAGGAGGTGC	54.4	
MMP9-F	CAGCCGACTTTTGTGGTCTTC	54.4	NM_004994.3
MMP9-R	GGTACAAGTATGCCTCTGCCA	54.4	
NKX2.5-F	AAGTGTGCGTCTGCCTTTCC	53.8	NM_001166175.2
NKX2.5-R	GCGCGCACAGCTCTTTCTTT	53.8	
SPARC1-F	CAGCCCCTTACTTAAAGGCCATA	55.3	NM_001128310.3
SPARC1-R	CAGCTGCAGTTCCCAAGAGA	53.8	
Spock2-F	GAAGCCACCCATTACGGTATGA	54.8	NM_001134434.1
Spock2-R	CTTGTGGATGCACGCAGTTT	51.8	

TBX5-F	GAGGTGGGATAGTTGGAGAGCAG	58.8	NM_000192.3
TBX5-R	GTCTTTTGCGTCAGGCTCCAG	56.3	
TIMP-F	CTGGGACACGCTTAGCATCA	53.8	NM_003254.3
TIMP-R	ACAGCGAGTGATCTTGCACT	51.8	
TLR4-F	CCAAGAACCTGGACCTGAGC	55.9	NM_003266.4
TLR4-R	TGTCTGGATTTCACACCTGGA	52.4	
TNF-F	GTAGCCCACGTCGTAGCAAA	53.8	NM_000594.4
TNF-R	TTGAGATCCATGCCGTTGGC	53.8	

Table S2. The mouse gene sequences of qPCR. All the primer sequences have validated the function through preliminary RT-PCR.

Primer ID	Sequences (5'→3')	TM (°C)	Product
GAPDH-F	AAGAGGGATGCTGCCCTTAC	53.8	NM_001289726.1
GAPDH-R	TACGGCCAAATCCGTTCACA	51.8	
HMGB1-F	ATAGGAAACTGCGGCCTCTC	53.8	NM_001313894.1
HMGB1-R	CCCATGTTTAGTTGATTTTCCAGC	54	
HSP-60-F	GGCCCAGGAACCAGCGTAG	57.6	NM_001356512.1
HSP-60-R	TGGGTAGTCGAAGCATTTCAGAG	55.3	
IL-1α-F	CGCTTGAGTCGGCAAAGAAT	52.4	NM_010548.2
IL-1α-R	TGGCAGAACTGTAGTCTTCGT	52.4	
S100A1-F	GGGAAAGACGACTAAAAGACAGG	55.3	NM_011309.3
S100A1-R	ATTTCAGCAGCACACGGTTG	51.8	
S100A8-F	ACAAGGAAATCACCATGCCC	51.8	NM_013650.2
S100A8-R	TTATATTCTGCACAAACTGAGGACA	52.8	
S100A9-F	TTTAGCCTTGAAGAGCAAGAAGA	51.7	NM_001281852.1
S100A9-R	AAGGTGTCGATGATGGTGGTT	52.4	

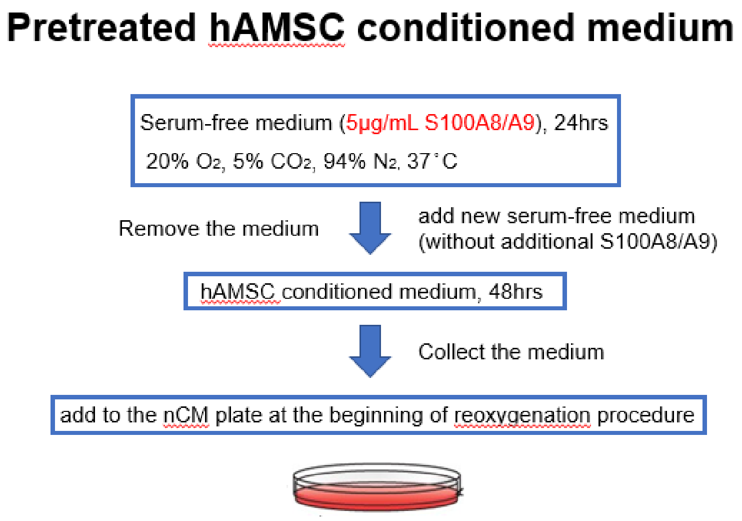

Figure S1. The flow scheme of S100A8/A9 pretreated hAMSC conditioned medium collected procedure.

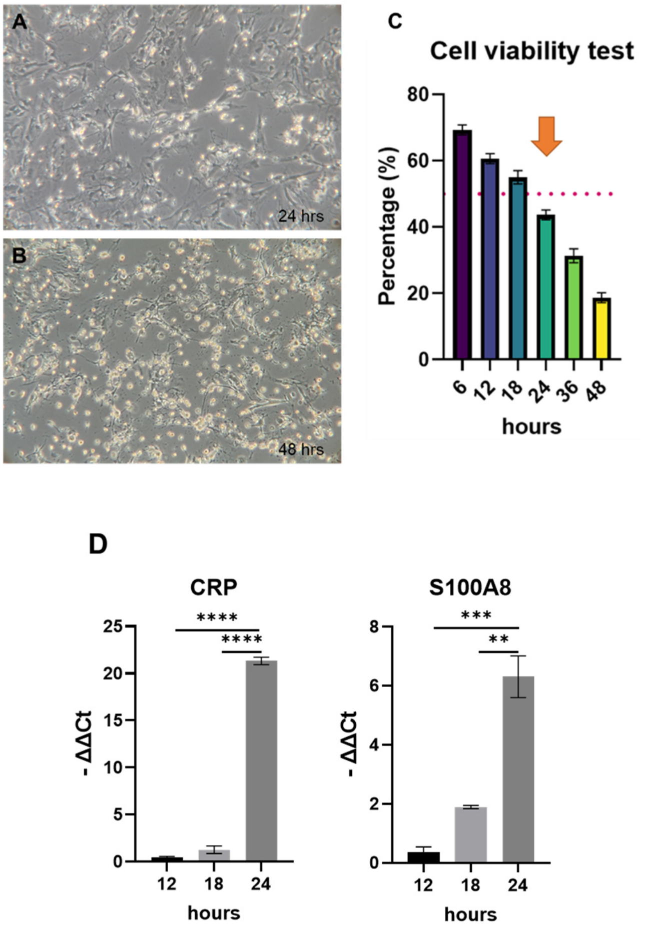

Figure S2. Determination of hypoxia condition through cell viability test and qPCR. (**A**,**B**) The cell viability images of neonatal mouse cardiomyocytes after 24 and 48 hours hypoxic treatment under microscopic observation. (**C**) The quantification of cell viability test of different hypoxic timing through trypan blue staining. (**D**) The comparison of cell injury related CRP and S100A8 gene expression after 12, 18, and 24 hours hypoxic condition through qPCR validation. The significance were calculated using one-way ANOVA. *$p < 0.05$, **$p < 0.01$, ***$p < 0.001$, ****$p < 0.0001$.
